# Mutilation of a Child by a Homœopathic Physician

**Published:** 1860-04

**Authors:** Hiram Nance

**Affiliations:** Lafayette, Ills.


### MUTILATION OF A CHILD BY A HOMŒOPATHIC PHYSICIAN

BY HIRAM NANCE, M. D., Lafayette, Ills.

Observing an article in the "Chicago Medical Journal," for January, bearing the above caption, inserting midwife instead of homœopathic physician, has led me to report the following case which come to my knowledge in the year 1855.

On the night of the 5th of May, being at Church, I was called out by an intelligent gentleman, a near neighbor of the patient, and requested to hurry on as fast as possible, with a positive injunction not to forget my obstetrical instruments, as he had been informed by the father of the patient that their use would be necessary. I inquired why such orders should be sent by a man making no pretensions to medical knowledge, and the messenger replied that they already had a physician, (a homœopath) but wanted me to hasten on and assist him out of a difficulty, from which he found it impossible to extricate himself, or save the life of patient or child.

Accordingly, with my instruments, I started for the house of the patient, telling the messenger at the same time that consultations and professional etiquette with homœopathic physicians were not tolerated in our school, but in a moderate degree; and not at all by myself individually. Telling him in plain and unequivocal terms, that when I arrived, should difficulties and perplexities present themselves in the case, that either I would take the case entirely into my own hands, or leave the house and let the Homœopath fight the affair out as best he could. When I arrived at the gate, a lady of my acquaintance met me, and said, "hurry, Doctor, I believe Mrs. D. will die!" I replied, Mrs. R., hav'nt you a physician? She said "yes, but he is good for nothing."

I went in, found Mrs. D. sitting up in bed, seemed awfully alarmed, face red, and turgid with blood. Rejoiced to see me, said "Oh! Doctor do help me, do! do!" I said Mrs. D., you have a physician, can't he give you the necessary assistance? She said, "No, I want you Dr. Nance."

This was the lady's first confinement, and her father had brought her home under the parental roof to remain until due time after her parturition. As the case had become alarming, her father, an old man of 65, had been admitted into the lying-in chamber to see his daughter, to sympathize, and if necessary, render any assistance manually to promote labor. I say the the old man sat by; and over in a corner, on a small couch, lay our *Homœopath.* I asked Mrs. D. and her husband if they desired me to take charge of the case; they replied in the affirmative; but the old man said he wanted "both Doctors, as the case required instrumental aid." I told him that I was not of the same school as Dr. L., and positively refused association in the practice of medicine in any of its branches with irregular bred physicians. The old man spoke up again, and said instruments must be used. I made no reply, but Mrs. D. and wife said, "We want you." This was enough. I immediately made an examination, found the head presenting in its first presentation, os uteri fully two-thirds dilated, and the head about engaging in the inferior straight; parts rigid, pains frequent, hard, but ineffectual. Observed some peculiarity about the scalp of the child which I could not account for. Inquired of the Homœopath (as he still reposed upon his couch in a *wakeful sleep* watching me) what could be the difficulty with the scalp; but he would make no reply. I told the ladies present, husband, and patient, that instruments were not required, but that in a short time by prudent management, I hoped she would be safely delivered; but I supposed the child from examination, to be dead.

I ordered a bandage, as the lady was quite plethoric, pulse full and heavy; opened a vein and bled nearly to syncope; this relaxed the system. I then gave a strong decoction of Secale Cornutum *pro re nata*, and in an hour's time my patient was safely delivered. Child breathed feebly for fifteen minutes, but never cried. On examination, found its scalp lacerated to an awful extent; it was torn into fragments, and a strong attempt had been made to break down the cranium and extract the child in fragments, but their obstetrical instruments were not manufactured sufficiently well to perform so grave an operation.

No one present had told me that any instrument had been used in the case; but now the matter was plain before me. I made inquiry what instrument had been used, and was told that the Homœopath and the old man had procured a large *strong awl*, such as is used in sewing leather, had bent it and then would hook it into the scalp, and both "*pull*" until the hold would give way; then try to break down the skull, but finally gave up in despair. *The child undoubtedly was killed by their manipulations.*

And now the matter was plain to my mind why I was urged to use instruments. Had I done so, those bruises and lacerations on the child's head would have been laid to me. As I did not I came out unscathed, and our "little pill" man was found consulting some of the first legal men in the State, fearing a suit for mal-practice would be commenced against him; or our Grand Jury would learn the facts in the case.

Suffice it to say, the patient got well, and the Homœopath made for parts westward.

